# Metabolomic signatures of the long-term exposure to air pollution and temperature

**DOI:** 10.1186/s12940-020-00683-x

**Published:** 2021-01-07

**Authors:** Feiby L. Nassan, Rachel S. Kelly, Anna Kosheleva, Petros Koutrakis, Pantel S. Vokonas, Jessica A. Lasky-Su, Joel D. Schwartz

**Affiliations:** 1 Department of Environmental Health, Harvard T. H. Chan School of Public Health, Landmark Center, Room 414C, 401 Park Dr, Boston, MA 02215 USA; 2 Channing Division of Network Medicine; Brigham and Women’s Hospital, Harvard Medical School, Boston, MA 02129 USA; 3 VA Normative Aging Study, VA Boston Healthcare System, School of Medicine and School of Public Health, Boston University, Boston, MA 02215 USA; 4 Department of Epidemiology, Harvard T. H. Chan School of Public Health, Boston, MA 02115 USA

**Keywords:** Metabolomics, Air pollution, Particulate matter, Temperature, Normative aging study (NAS)

## Abstract

**Background:**

Long-term exposures to air pollution has been reported to be associated with inflammation and oxidative stress. However, the underlying metabolic mechanisms remain poorly understood.

**Objectives:**

We aimed to determine the changes in the blood metabolome and thus the metabolic pathways associated with long-term exposure to outdoor air pollution and ambient temperature.

**Methods:**

We quantified metabolites using mass-spectrometry based global untargeted metabolomic profiling of plasma samples among men from the Normative Aging Study (NAS). We estimated the association between long-term exposure to PM_2.5_, NO_2_, O_3_, and temperature (annual average of central site monitors) with metabolites and their associated metabolic pathways. We used multivariable linear mixed-effect regression models (LMEM) while simultaneously adjusting for the four exposures and potential confounding and correcting for multiple testing. As a reduction method for the intercorrelated metabolites (outcome), we further used an independent component analysis (ICA) and conducted LMEM with the same exposures.

**Results:**

Men (*N* = 456) provided 648 blood samples between 2000 and 2016 in which 1158 metabolites were quantified. On average, men were 75.0 years and had an average body mass index of 27.7 kg/m^2^. Almost all men (97%) were not current smokers. The adjusted analysis showed statistically significant associations with several metabolites (58 metabolites with PM_2.5_, 15 metabolites with NO_2_, and 6 metabolites with temperature) while no metabolites were associated with O_3_. One out of five ICA factors (factor 2) was significantly associated with PM_2.5_. We identified eight perturbed metabolic pathways with long-term exposure to PM_2.5_ and temperature: glycerophospholipid, sphingolipid, glutathione, beta-alanine, propanoate, and purine metabolism, biosynthesis of unsaturated fatty acids, and taurine and hypotaurine metabolism. These pathways are related to inflammation, oxidative stress, immunity, and nucleic acid damage and repair.

**Conclusions:**

Using a global untargeted metabolomic approach, we identified several significant metabolites and metabolic pathways associated with long-term exposure to PM_2.5_, NO_2_ and temperature. This study is the largest metabolomics study of long-term air pollution, to date, the first study to report a metabolomic signature of long-term temperature exposure, and the first to use ICA in the analysis of both.

**Supplementary Information:**

The online version contains supplementary material available at 10.1186/s12940-020-00683-x.

## Highlights

1. Long-term air pollution exposure is associated with adverse outcomes.
2. The blood metabolome is a powerful tool for mechanistic understanding.
3. We identified metabolites and metabolic pathways that were perturbed with long-term exposure to PM_2.5_ and temperature.
4. Similar to short-term exposure, the perturbed pathways with long-term exposure are related to inflammation and oxidative stress, immunity, and nucleic acid damage and repair.

## Introduction

Long-term exposures to air pollution [1–3] and temperature [4] have been reported to be associated with oxidative stress and inflammation. These biological processes are linked to mortality [5, 6] and several adverse health effects such as pulmonary [7], cardiovascular [8], and neurological diseases [9]. However, the affected metabolic mechanisms linking these exposures and phenotypes remain poorly understood. The metabolome is a collection of biologically active chemicals derived from the combination of endogenous processes and exogenous exposures [10]. Metabolomic profiling of blood is a powerful tool to increase mechanistic understanding. Because some ambient air pollutants e.g., fine particles, have been reported to enter the bloodstream directly from the lungs, a blood metabolomic signature associated with ambient air pollution exposure is plausible [11]. Even larger particles that cannot cross the pulmonary epithelium and ambient temperature can induce inflammation in the lungs and trigger a systemic response observed in the blood [12, 13].

Recently a few studies [14–19], including one (currently under consideration for publication elsewhere) based within the cohort studied here (The Normative Aging Study, NAS), have examined the metabolomic signatures of short-term exposure (days) to air pollution and, less frequently, short-term temperature [20]. However, to date, only three studies focused on long-term exposure to air pollution [21–23] and no human studies have examined the effects of long-term exposure to temperature, thus the mechanistic understanding is still lacking. Furthermore, a few studies have employed methods to capture the high dimensionality of the data and account for the intercorrelation of the metabolites within co-regulated pathways.

Furthermore, a key issue in the existing studies of temperature and mortality is that they do not merely identify increased deaths at the extremes of temperature [24]. They generally report U-shaped relationships with a minimum mortality temperature, typically in the range of 15–20 °C, with increased death rates at any other temperature [24]. For example, there is clearly increased mortality in Boston at 25 °C compared to the minimum mortality temperature [24]. Furthermore, similar associations of non-extreme temperature exposure with other adverse health outcomes other than mortality were reported [25]. Hence it is critical to identify mechanisms by which temperature may have these effects. Changes in the metabolome are one such possible mechanism.

Therefore, we aimed in this study to determine the blood metabolomic signature of long-term exposure to outdoor air pollution and temperature, while using novel statistical methods e.g., independent component analysis (ICA) to reduce the dimensions of the intercorrelated data. Using Ultrahigh Performance Liquid Chromatography Coupled Tandem Mass Spectroscopy (UPLC-MS/MS) based plasma untargeted metabolomic profiling of a large cohort of men (NAS), we hypothesized that long-term (annual) exposure to air pollution and temperature are associated with perturbed biological pathways and corresponding blood metabolomic signatures. We further tested if there was an effect modification by metabolic conditions like obesity or diabetes.

## Materials and methods

### Participants and study design

The NAS was established in 1963 in Boston, Massachusetts. It is a longitudinal aging study among men. Men (*N* = 2280), 21–80 years old and free of known chronic diseases were enrolled in the NAS between 1963 and 1970 and have been followed since [26]. The review boards of Harvard T.H. Chan School of Public Health and the Department of Veterans Affairs approved the NAS. All participants provided written informed consent. During the follow-up visits, participants periodically self-reported information about their medical history, dietary intake, and other health-related history. In addition, participants had physical examinations and laboratory tests every 3–5 years. At every visit, a 7 ml fasting venous blood sample was collected in a trace metal-free tube containing ethylenediaminetetraacetic acid. The samples were spun for 15 min at 3000 revolutions per minute (RPM). Serum and plasma samples were placed in 1.8 ml Nunc tubes for long-term storage at − 80 °C.

For the current analysis, we considered 464 men who provided 659 blood samples between 2000 and 2016 in which blood metabolomics profiling was performed. Blood samples collected prior to 2000 were not suitable for metabolomic profiling due to their storage conditions. Because only eight men were non-white, we restricted our final sample size to 456 white men who provided 648 blood samples.

### Quantification of air pollution and temperature (exposure)

In the current study, we focused on long-term exposures to air pollution and temperature as measured by the annual average (365 days) of the levels before the visit for each blood draw. The air pollutants were particulate matter ≤2.5 μm (PM_2.5_) and gaseous pollutants: nitrogen dioxide (NO_2_) and ozone (O_3_). All exposures were measured near downtown Boston, MA, at a fixed monitoring site at the Harvard University Countway Library and ~ 1 km from the NAS examination center. Because study participants lived in the greater Boston area with a median distance of ~ 20.8 km from the examination center [27], we considered the ambient air pollutant levels as participants’ long-term exposure surrogates. Levels of PM_2.5_ (μg/m^3^) were measured hourly using a tapered element oscillation microbalance (Model 1400A, Rupprecht and Pastashnick) [28]. Hourly NO_2_ and O_3_ levels (part per billion (ppb)) were measured at local Massachusetts Department of Environmental Protection monitoring sites in the greater Boston area and were averaged based on data from all available sites. We obtained daily temperature and relative humidity data from the national weather service station at Logan airport (Boston, MA), located ~ 12 km from the NAS examination site. Study participants lived throughout the metropolitan area. Therefore, we considered the monitored temperature as surrogates of their long-term exposures.

### Metabolomic profiling (outcomes)

All samples were sent to the lab and analyzed at the same timepoint. Metabolon Inc. (Durham, NC, USA) conducted metabolomic profiling. To enable the broadest coverage of the metabolome, untargeted high-resolution UPLC-MS/MS was used. The methods were previously described in detail [29]. In brief, the four platforms were: (1) UPLC-MS/MS under positive ionization for early eluting metabolites, (2) UPLC-MS/MS under positive ionization for late eluting metabolites, (3) UPLC-MS/MS under negative ionization, and (4) UPLC-MS/MS, polar (negative ionization) platform. The samples were randomized for profiling and in-house standards and internal QCs were used to account for potential batch effects across the runs [29]. Metabolites were identified by their mass-to-charge ratio, retention time, and through a comparison to a library of purified known standards. Metabolites were quantified using area-under-the-curve (AUC) of the peak and processed according to our in-house standard quality control pipeline [30, 31] which retains the maximum number of metabolites, including those with a high level of missingness as these may represent biologically important markers of exposure in a subset of the population, while excluding statistically uninformative metabolites. Missing values were imputed with half of the minimum observed level for a given metabolite [31]. Then, metabolite levels were log-transformed and *pareto* scaled. A total of 1301 metabolites were profiled including 143 metabolites with an interquartile range of zero that we considered uninformative and hence excluded. This left 1158 metabolites available for the current analysis.

### Statistical analysis

We calculated descriptive statistics to summarize demographics and lifestyle factors for the study participants during the first visit as well as all visits. We examined whether the annual averages of the levels of PM_2.5_, NO_2_, O_3_, and temperature prior to visit were associated with changes in the levels of the 1158 metabolites. To account for the correlation of repeated measures within the same participant, we used linear mixed-effect regression models (LMEM) with random participant-specific intercepts. We first conducted generalized additive mixed models (GAMM) with penalized spline for temperature to check if linearity would fit better, then we used LMEM as our final models because temperature fit better as a linear term. We modeled the outcomes and the exposures as continuous variables. We simultaneously adjusted for PM_2.5_, NO_2_, O_3_, and temperature (multi-pollutant models) as shown below. We further adjusted for potential confounders and predictors of the outcomes including age (years), body mass index (kg/m^2^), socioeconomic status (annual income and years of education), cigarette pack-years, alcohol intake (< or ≥ 2 drinks per day), season at blood draw (warm/cold), and relative humidity.

*Multi− pollutant models*:

$$ E\left({Y}_{ij}\right)={\beta}_0+{\mu}_i+{\beta}_1\ {PM}_{2.5 ij}+{\beta}_2{NO}_{2 ij}+{\beta}_3{O}_{3 ij}+{\beta}_4\ {Temperature}_{ij}+{\beta}_{5-n}\ {Covariates}_{ij} $$

where *Y*_*ij*_ is the metabolome level of subject i at visit j, *β*_0_ is the fixed intercept, *μ*_*i*_ is the random intercept for subject i, and the annual averages of the air pollutants and temperature for subject i at visit j.

To correct for multiple testing while also accounting for the highly-correlated metabolites that are closely connected through interlinked biological pathways, we conducted the number of the effective tests approach (ENT) [32, 33]. This method determines the number of principal components required to explain a given percentage of the variance in the data (i.e., the number of effective/independent tests). We calculated the adjusted *p*-value threshold as α/*m* where α is the nominal *p*-value of 0.05, and *m* is the number of principal components. We used thresholds of 95% (ENT95%) and 99% (ENT99%) variance explained and further divided it by four (investigated exposures). We finally tested for effect modification by type II diabetes and obesity (BMI ≥30 kg/m^2^) for the four exposures by adding interaction terms for every metabolite.

In order to explore metabolomic profiles rather than individual metabolites, we also applied independent component analysis (ICA) [34] as an unsupervised technique to reduce the dimension of the highly-correlated metabolites into five independent ICA-factors. The more commonly used principal component analysis (PCA) is a tool for dividing multiple correlated variables into uncorrelated factors. If the variables are normally distributed then uncorrelated factors are also independent, however that is not true for non-normally distributed variables such as metabolites here. In contrast, ICA is a computational method for separating multivariate signal into additive factors that are maximally independent that assumes non-normal signals [34]. There is an attached weight for each metabolite to determine its individual contributing weight to each ICA-factor. We then conducted LMEM where those five ICA independent factors were the outcomes while exposures and covariates remained as above. We further corrected for multiple testing in the ICA models by applying Benjamini-Hochberg false discovery rate (FDR), set the false positive threshold as 5% [35], and further accounted for the four investigated exposures (PM_2.5_, NO_2_, O_3_, and temperature).

As a sensitivity analysis, we conducted LMEM models for each single pollutant or temperature at a time (single-pollutant models) as shown below, while adjusting for the covariates as above (but not other exposures). The single-pollutant models for air pollutants were still simultaneously adjusted for temperature. Analyses were conducted using R version 4.0.1 and SAS version 9.4 (SAS Institute Inc., Cary, NC, USA).

Single − pollutant models:

$$ E\left({Y}_{ij}\right)={\beta}_0+{\mu}_i+{\beta}_1\ {PM}_{2.5 ij}+{\beta}_2\ {Temperature}_{ij}+{\beta}_{3-n}\ {Covariates}_{ij} $$

$$ E\left({Y}_{ij}\right)={\beta}_0+{\mu}_i+{\beta}_1\ {NO}_{2 ij}+{\beta}_2\ {Temperature}_{ij}+{\beta}_{3-n}\ {Covariates}_{ij} $$

$$ E\left({Y}_{ij}\right)={\beta}_0+{\mu}_i+{\beta}_1\ {O}_{3 ij}+{\beta}_2\ {Temperature}_{ij}+{\beta}_{3-n}\ {Covariates}_{ij} $$

$$ E\left({Y}_{ij}\right)={\beta}_0+{\mu}_i+{\beta}_1\ {Temperature}_{ij}+{\beta}_{3-n}\ {Covariates}_{ij} $$

As an exploratory analysis to better understand the significant metabolites, we conducted pathway analysis for the significant metabolites (at *p*-value < 0.01) from the multi-pollutant model and the 100 metabolites that had the greatest weighting on the significant ICA-factor(s). To do that, we used the ‘Pathway Analysis’ functionality in MetaboAnalyst 4.0, that accounts for both over-representation (i.e., how many significant metabolites fall within a given pathway) and pathway topology (i.e., how important those metabolites are to that pathway) [36] using Human Metabolome Database (HMDB), and Kyoto Encyclopedia of Genes and Genomes (KEGG) databases. The structure of metabolic pathways provides information on the complex relationships between molecules (activation, inhibition, or reaction, etc.). The pathway topology analyses incorporates this information by using two well-established node centrality measures to estimate the importance of a given metabolite within a pathway 1) betweenness centrality which focuses on the position of the metabolite relative to overall pathway structure and 2) degree centrality which focuses on how connected that metabolite is to other metabolites within the pathway [37]. The importance of each metabolite is defined as the percentage in relation to the total pathway importance, and the pathway impact is defined as the cumulative percentage from the matched metabolites [37].

We considered statistical significance for the pathways at a nominal *p*-value ≤0.1 and considered additional noteworthy pathways if the impact score was ≥0.5 while nominal *p*-value < 0.3.

## Results

Participants (*N* = 456) provided 648 blood samples in which 1158 distinct metabolites were quantified and passed our in-house QC pipeline [30, 31]. Approximately 64% of the participants provided one blood sample, 31% provided two samples, and 6% provided three samples. The measured metabolites constituted lipids (39%), followed by metabolites of un-identified origin (19%), amino acids (17%), xenobiotics (12%), nucleotides (3%), cofactor and vitamins (3%), carbohydrates (2%), peptides (2%), partially characterized molecules (2%), and energy metabolites (1%). At the initial visit, men’s mean age was ~ 75.0 years, mean body mass index was 27.7 kg/m^2^, mean annual income was 8.61 thousand of 1965 $US, and participants had received a mean of 15.1 years of education (Table 1). Almost all participants (97%) were not current smokers. Most of the participants (79%) consumed < 2 alcoholic drinks/day. Approximately 60% of the visits occurred during the warm season (April to September). Average values of air pollution and temperature over the follow-up period remained stable across the 365-day exposure window (Table 1).

**Table 1:** Characteristics of the study population in the Normative Aging Study (2000 to 2016)

Demographic Characteristics	At first visit (2000 to 2013) (*N* = 456)	All visits (2000 to 2016) (*N* = 648)
Age, Mean (SD)	75.0 (6.70)	76.0 (6.68)
Body mass index (kg/m^2^), Mean (SD)	27.7 (4.19)	27.7 (4.27)
Years of education, Mean (SD)	15.1 (3.00)	15.0 (3.00)
Baseline annual income, thousands of 1965 $US, Mean (SD)	8.61 (3.77)	8.64 (3.78)
Smoking		
Never, N (%)	128 (28)	190 (29)
Current, N (%)	14 (3)	21 (3)
Former, N (%)	314 (69)	437 (67)
Pack-year smoked (years), Mean (SD)	21.4 (25.2)	20.95 (25.36)
Season		
Cold: October–March, N (%)	189 (41)	259 (40)
Warm: April–September, N (%)	267 (59)	389 (60)
Alcohol consumption (≥2 drinks per day), N (%)	95 (21)	132 (20)
Mean (SD), (IQR) of the exposure levels		
Average of 365 days of PM_2.5_ (μg/m3)	10.4 (1.28), (2.49)	10.1 (1.31), (2.12)
Average of 365 days of NO_2_ (ppb)	19.1 (2.40), (4.52)	18.4 (2.35), (4.00)
Average of 365 days of O_3_ (ppb)	23.7 (1.06), (1.39)	24.0 (1.12), (1.45)
Average of 365 days of Temperature (°C)	11.0 (0.64), (1.00)	11.0 (0.60), (0.89)

*Abbreviations*: *N* Number of participants or visits, *SD* Standard deviation, *ppb* is part per billion, *Kg* Kilogram, *m* Meters, *C* Celsius, *IQR* Interquartile range

**Fig. 1:**
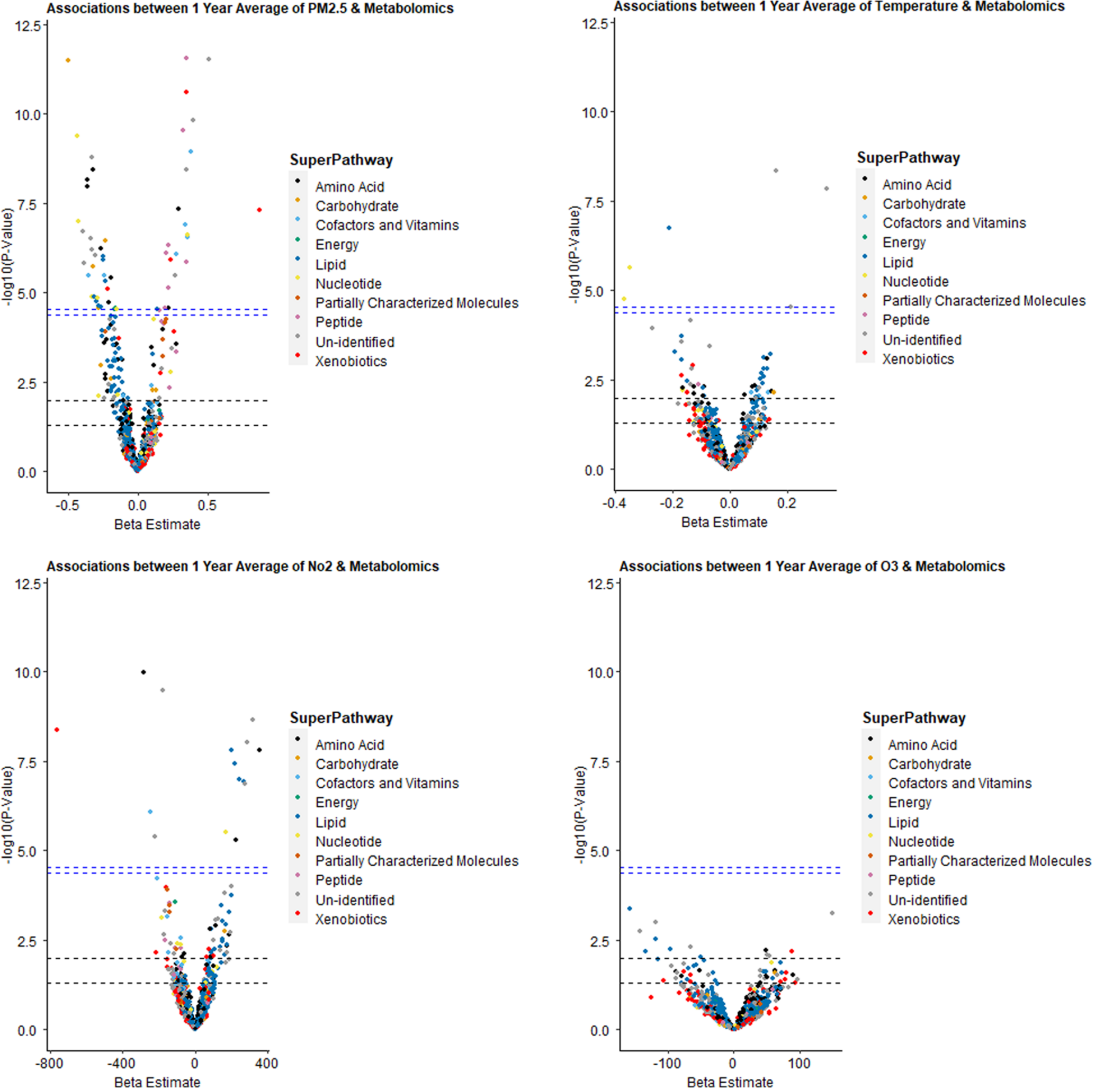
Volcano Plots presenting the adjusted associations between long-term exposures to air pollutants and temperature with metabolomics (multi-pollutant models). These models were linear mixed-effect regression models (LMEM) with random participant-specific intercepts and simultaneously adjusted for PM_2.5_, NO_2_, O_3_, and temperature (multi-pollutant models) for the same exposure window. All models were adjusted for age (years), body mass index (kg/m^2^), cigarette pack-years, alcohol intake (< or ≥ 2 drinks per day), socioeconomic status (income payment and years of education), season (warm/cold), and relative humidity. The transverse dashed lines represent different statistical significance levels of *p*-values (from lower to upper): 0.05, 0.01, ENT95%, and ENT99%. Note the different scale of the X axes. Abbreviations: ENT: Effective/independent number of tests; log10, logarithmic base 10; PM_2.5_, particulate matter ≤2.5 μm; NO_2_, nitrogen dioxide; and O_3_, ozone

After adjusting for potential confounding and correcting for multiple testing in the multi-pollutant models, PM_2.5_, NO_2_, and temperature showed statistically significant associations with several metabolites (58 metabolites with PM_2.5_, 15 metabolites with NO_2_, and 6 metabolites with temperature) out of 1158 metabolites while no metabolites were associated with O_3_ (Fig. 1, Tables 2 and 3, Supplemental Table 1). It is worth mentioning that several significant metabolites were unidentified at the time of this analysis, and annotation is pending. On the other hand, heptanoate (7:0), inosine, and guanosine were among the identified ones, for example with ambient temperature exposure, with negative associations (Table 3). In the ICA analysis, in the multi-pollutant models, PM_2.5_ was significantly associated with ICA-factor 2 only but not with NO_2_ or O_3_ (Supplemental Table 2). Many of the most contributing metabolites (highest 100) to ICA-factor 2 were also individually significant with exposures of interest in the multi-pollutant models (Tables 2 and 3, Supplemental Table 1). We did not observe an effect modification by diabetes or obesity for any of the examined exposures on any of the metabolites examined at the ENT95% level. Results were similar in the sensitivity analysis of the single-pollutant models, but in the latter analysis a larger number of metabolites were significantly associated with each outcome (Supplemental Figure 1 and Supplemental Table 3).

**Table 2:** The significant adjusted associations between long-term (annual) exposure to PM_2.5_ with metabolomics (multi-pollutant models) at ENT95% significance level

BIOCHEMICAL	Super-Pathway	Sub-Pathway	Beta (SE) for individual metabolites	***P***-value for individual metabolites	ICA_factor2 Weight	ICA_factor2 Rank
Aspartate	Amino Acid	Alanine and Aspartate Metabolism	− 0.203 (0.046)	1.93E-05	0.392	57
Cys-Gly, Oxidized	Amino Acid	Glutathione Metabolism	−0.357 (0.060)	1.10E-08	0.527	19
Cysteinylglycine Disulfide^*^	Amino Acid	Glutathione Metabolism	−0.316 (0.051)	4.00E-09	0.434	43
Cysteinylglycine	Amino Acid	Glutathione Metabolism	−0.361 (0.059)	7.00E-09	0.266	115
Sarcosine	Amino Acid	Glycine, Serine and Threonine Metabolism	0.293 (0.051)	4.60E-08	0.094	291
Alpha-Hydroxyisocaproate	Amino Acid	Leucine, Isoleucine and Valine Metabolism	−0.266 (0.051)	5.69E-07	0.483	27
Taurine	Amino Acid	Methionine, Cysteine, SAM and Taurine Metabolism	−0.190 (0.040)	3.79E-06	0.423	45
Cysteine S-Sulfate	Amino Acid	Methionine, Cysteine, SAM and Taurine Metabolism	−0.580 (0.075)	6.76E-13	0.412	50
5-Methylthioadenosine (MTA)	Amino Acid	Polyamine Metabolism	0.222 (0.052)	2.77E-05	−0.277	108
Maltose	Carbohydrate	Glycogen Metabolism	−0.321 (0.065)	1.90E-06	0.536	17
3-Phosphoglycerate	Carbohydrate	Glycolysis, Gluconeogenesis, and Pyruvate Metabolism	−0.499 (0.067)	3.33E-12	0.856	2
Lactate	Carbohydrate	Glycolysis, Gluconeogenesis, and Pyruvate Metabolism	−0.233 (0.044)	3.54E-07	0.448	39
Ribitol	Carbohydrate	Pentose Metabolism	−0.278 (0.065)	2.68E-05	0.204	152
Heme	Cofactors and Vitamins	Hemoglobin and Porphyrin Metabolism	−0.348 (0.073)	3.34E-06	0.599	10
Bilirubin (E,Z Or Z,E)^*^	Cofactors and Vitamins	Hemoglobin and Porphyrin Metabolism	0.354 (0.066)	2.93E-07	−0.327	79
Bilirubin (E,E)^*^	Cofactors and Vitamins	Hemoglobin and Porphyrin Metabolism	0.343 (0.062)	1.30E-07	−0.260	118
Bilirubin (Z,Z)	Cofactors and Vitamins	Hemoglobin and Porphyrin Metabolism	0.381 (0.059)	1.00E-09	−0.117	243
Nicotinamide	Cofactors and Vitamins	Nicotinate and Nicotinamide Metabolism	−0.237 (0.049)	3.27E-06	0.515	22
Alpha-Tocopherol	Cofactors and Vitamins	Tocopherol Metabolism	0.279 (0.055)	8.57E-07	−0.121	233
Malate	Energy	TCA Cycle	−0.164 (0.038)	2.93E-05	0.311	90
Succinate	Energy	TCA Cycle	−0.161 (0.037)	2.72E-05	0.249	127
1-Stearoyl-GPS (18:0)^*^	Lipid	Lysophospholipid	−0.31 (0.069)	1.30E-05	0.640	7
1-Arachidonoyl-GPA (20:4)	Lipid	Lysophospholipid	−0.282 (0.065)	2.40E-05	0.462	34
1,2-Dipalmitoyl-GPE (16:0/16:0)^*^	Lipid	Phosphatidylethanolamine (PE)	−0.299 (0.068)	1.79E-05	0.406	52
1-Stearoyl-2-Oleoyl-GPS (18:0/18:1)	Lipid	Phosphatidylserine (PS)	−0.264 (0.061)	2.55E-05	0.640	8
Phosphoethanolamine	Lipid	Phospholipid Metabolism	−0.228 (0.049)	4.92E-06	0.552	14
Sphinganine	Lipid	Sphingolipid Synthesis	−0.249 (0.049)	9.96E-07	0.530	18
Sphingomyelin (D18:2/18:1)^*^	Lipid	Sphingomyelins	0.137 (0.032)	2.88E-05	−0.066	423
Sphingosine	Lipid	Sphingosines	−0.249 (0.049)	1.17E-06	0.562	13
Inosine 5′-Monophosphate (IMP)	Nucleotide	Purine Metabolism, (Hypo)Xanthine/Inosine containing	−0.423 (0.076)	1.02E-07	0.799	3
Hypoxanthine	Nucleotide	Purine Metabolism, (Hypo)Xanthine/Inosine containing	−0.327 (0.073)	1.33E-05	0.755	5
Allantoin	Nucleotide	Purine Metabolism, (Hypo)Xanthine/Inosine containing	−0.229 (0.043)	3.45E-07	0.012	1007
Adenosine 5′-Monophosphate (AMP)	Nucleotide	Purine Metabolism, Adenine containing	−0.433 (0.066)	4.16E-10	0.797	4
Dihydroorotate	Nucleotide	Pyrimidine Metabolism, Orotate containing	0.356 (0.066)	2.41E-07	−0.279	106
Uracil	Nucleotide	Pyrimidine Metabolism, Uracil containing	−0.284 (0.064)	1.40E-05	0.399	56
Beta-Alanine	Nucleotide	Pyrimidine Metabolism, Uracil containing	−0.149 (0.035)	2.87E-05	0.214	147
Gamma-Glutamylmethionine	Peptide	Gamma-glutamyl Amino Acid	0.324 (0.049)	2.84E-10	−0.384	60
Gamma-Glutamylalanine	Peptide	Gamma-glutamyl Amino Acid	0.347 (0.07)	1.41E-06	−0.360	65
Gamma-Glutamylglycine	Peptide	Gamma-glutamyl Amino Acid	0.35 (0.047)	2.76E-12	−0.290	100
Gamma-Glutamylglutamine	Peptide	Gamma-glutamyl Amino Acid	0.22 (0.042)	4.59E-07	−0.250	125
Gamma-Glutamylvaline	Peptide	Gamma-glutamyl Amino Acid	0.216 (0.047)	7.33E-06	−0.229	138
Gamma-Glutamylisoleucine^*^	Peptide	Gamma-glutamyl Amino Acid	0.197 (0.046)	2.64E-05	−0.205	151
Gamma-Glutamyl-Alpha-Lysine	Peptide	Gamma-glutamyl Amino Acid	0.207 (0.04)	7.56E-07	−0.192	162
Gamma-Glutamylthreonine	Peptide	Gamma-glutamyl Amino Acid	0.152 (0.036)	3.03E-05	−0.077	351
Iminodiacetate (IDA)	Xenobiotics	Chemical	0.352 (0.049)	2.51E-11	−0.125	227
Hepes	Xenobiotics	Chemical	0.874 (0.154)	4.90E-08	0.062	447
Tartronate (Hydroxymalonate)	Xenobiotics	Food Component/Plant	0.238 (0.047)	1.19E-06	−0.442	41
Ergothioneine	Xenobiotics	Food Component/Plant	−0.213 (0.046)	7.84E-06	0.268	112
X - 24,970	Un-identified	–	−0.389 (0.072)	2.01E-07	0.360	63
X - 24,307	Un-identified	–	0.512 (0.068)	3.06E-12	−0.340	74
X - 23,739	Un-identified	–	0.266 (0.056)	3.36E-06	− 0.337	76
X - 12,104	Un-identified	–	0.349 (0.056)	4.00E-09	−0.319	84
X - 11,632	Un-identified	–	−0.329 (0.052)	2.00E-09	0.318	85
X - 13,684	Un-identified	–	−0.383 (0.077)	1.55E-06	0.317	86
X - 24,306	Un-identified	–	0.397 (0.059)	1.57E-10	−0.165	184
X - 24,431	Un-identified	–	−0.334 (0.063)	3.10E-07	0.160	190
X - 14,904	Un-identified	–	−0.327 (0.063)	6.26E-07	−0.119	237
X - 10,458	Un-identified	–	−0.303 (0.059)	8.90E-07	0.022	865

These models were linear mixed-effect regression models (LMEM) with random participant-specific intercepts and simultaneously adjusted for PM_2.5_, NO_2_, O_3_, and temperature (multi-pollutant models) for the same exposure window

Beta (SE) and *p*-values presented are from individual metabolites analysis

All models were adjusted for age (years), body mass index (kg/m^2^), cigarette pack-years, alcohol intake (< or ≥ 2 drinks per day), socioeconomic status (income payment and years of education), season (warm/cold), and relative humidity

Significant metabolites at ENT95% significance level only are presented

ICA_factor2 rank represents the rank of the corresponding metabolite contributing to factor 2 from the independent component analysis (ICA). Higher rank (and higher weights) means higher contribution to factor 2 of the ICA. We show alongside the weights and ranks of each metabolite that contributes to factor 2 from the ICA only because it was the only significant factor

Range of facto2- ICA rank is from 1 to 1158 i.e., number of examined metabolites. Range of facto2- ICA weight is from − 1 to 1

The metabolites with an X-XXXX format are unknown (to date Metabolon has not been able to name the metabolite) but reproducible (Metabolon is reliably able to characterize this metabolite in multiple samples and studies)

*Abbreviations*: *ENT* Effective/independent number of tests, *PM*_*2.5*_ Particulate matter ≤2.5 μm, *NO*_*2*_ Nitrogen dioxide, and *O*_*3*_ Ozone, *ICA* Independent component analysis, *SE* Standard error

*Indicates a compound that has not been confirmed based on a standard, but Metabolon is confident in its identity based on a subset of these analytical parameters

**Table 3:** The significant adjusted associations between long-term exposure windows to temperature with metabolomics (multi-pollutant models) at ENT95% significance level

Biochemical	Super-Pathway	Sub-Pathway	Beta (SE) for individual metabolites	***P***-value for individual metabolites	ICA_factor2 Weight	ICA_factor2 Rank
Heptanoate (7:0)	Lipid	Medium Chain Fatty Acid	−0.209 (0.039)	1.87E-07	−0.282	104
Inosine	Nucleotide	Purine Metabolism, (Hypo)Xanthine/Inosine containing	−0.368 (0.083)	1.71E-05	0.892	1
Guanosine	Nucleotide	Purine Metabolism, Guanine containing	−0.348 (0.071)	2.23E-06	0.329	78
X - 24,970	Un-identified	–	0.34 (0.057)	1.50E-08	0.360	63
X - 24,431	Un-identified	–	0.215 (0.05)	2.91E-05	0.160	190
X - 23,636	Un-identified	–	0.161 (0.026)	5.00E-09	0.141	214

These models were linear mixed-effect regression models (LMEM) with random participant-specific intercepts and simultaneously adjusted for PM_2.5_, NO_2_, O_3_, and temperature (multi-pollutant models) for the same exposure window

Beta (SE) and *p*-values presented are from individual metabolites analysis

All models were adjusted for age (years), body mass index (kg/m^2^), cigarette pack-years, alcohol intake (< or ≥ 2 drinks per day), socioeconomic status (income payment and years of education), season (warm/cold), and relative humidity

Significant metabolites at ENT95% significance level only are presented

ICA_factor2 rank represents the rank of the corresponding metabolite contributing to factor 2 from the independent component analysis (ICA). Higher rank (and higher weights) means higher contribution to factor 2 of the ICA. We show alongside the weights and ranks of each metabolite that contributes to factor 2 from the ICA only because it was the only significant factor

Range of facto2- ICA rank is from 1 to 1158 i.e., number of examined metabolites. Range of facto2- ICA weight is from −1 to 1

The metabolites with an X-XXXX format are unknown (to date Metabolon has not been able to name the metabolite) but reproducible (Metabolon is reliably able to characterize this metabolite in multiple samples and studies)

*Abbreviations*: *ENT* Effective/independent number of tests, *PM*_*2.5*_ Particulate matter ≤2.5 μm, *NO*_*2*_ Nitrogen dioxide, and *O*_*3*_ Ozone, *ICA* Independent component analysis, *SE* Standard error, *NS* Not significant at the ENT95% level

**Fig. 2:**
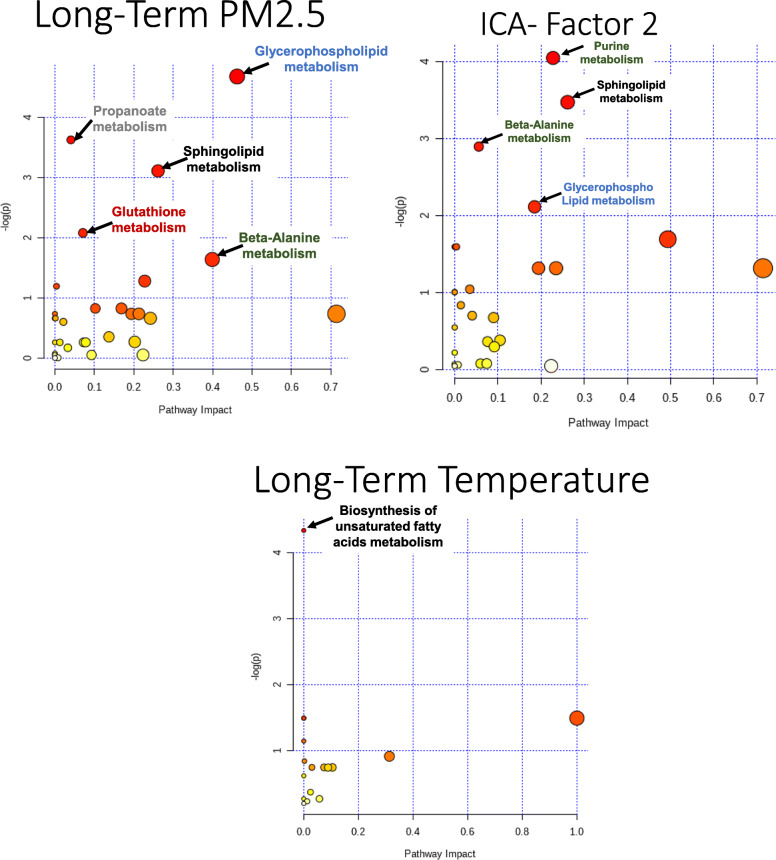
Metabolic Pathways identified as enriched among the metabolites significantly associated with **a** Long-term exposure to PM_2.5_; **b** top 100 metabolites loading on to ICA-Factor 2; and **c** Long-term exposure to temperature. Pathway analysis conducted using Metaboanalyst [36] is based on both over enrichment analysis, i.e. how many significant metabolites fall within a given pathway, and pathway topology analysis, i.e. how important those metabolites are to that pathway. The figure shows the –log (natural logarithm) of the enrichment *p*-value on the x-axis, and the impact score on the y-axis which is based on the cumulative importance of all the significant metabolites within the pathway. The size of each bullet represents the impact value. The color of each bullet represents the significance of the enrichment. Abbreviations: PM_2.5_, particulate matter ≤2.5 μm; ICA, independent component analysis

In the exploratory pathway analysis of the significant metabolites for each exposure, long-term exposure to PM_2.5_ was associated with five metabolic pathways including: glycerophospholipid (*p*-value = 0.009), propanoate (*p*-value = 0.03), sphingolipid (*p*-value = 0.04), and glutathione (*p*-value = 0.12) metabolism (Fig. 2 and Supplemental Table 4). Long-term exposure to NO_2_ was not significantly associated with any metabolic pathways. Long-term exposure to temperature was associated with biosynthesis of unsaturated fatty acids (*p*-value = 0.01). The pathways that were associated with the 100 highest contributing metabolites to ICA-factor 2 were purine (*p*-value = 0.02), sphingolipid (*p*-value = 0.03), beta-alanine (*p*-value = 0.06), and glycerophospholipid (*p*-value = 0.12) metabolism. Taurine and hypotaurine metabolism was also noteworthy due to high impact score of 0.71 while *p*-value was 0.27.

## Discussion

Using an untargeted metabolomic approach, we observed several significant associations between long-term air pollutants (PM_2.5_ and NO_2_) and temperature with several blood metabolites. We identified eight metabolic pathways perturbed with long-term exposure to PM_2.5_ and temperature: glycerophospholipid, sphingolipid, glutathione, beta-alanine, propanoate, purine metabolisms, biosynthesis of unsaturated fatty acids, and possibly taurine and hypotaurine metabolism. In this study, we assessed the largest number of metabolites using an untargeted approach and the largest sample size to date in studies of metabolite association with long-term air pollution. This is also the first study to report a metabolomic signature of long-term temperature exposure, and the first to use ICA in the analysis of both. There was an overlap between the perturbed pathways when we used different statistical methods i.e., regression models and ICA. Previously some studies [38, 39] used PCA to analyze metabolomics data which produces uncorrelated factors, a necessary but insufficient condition for independence. In comparison ICA is a computational method to separate multivariate signals into additive maximally independent factors and assumes non-normal signals [34].

For example, with ambient temperature, heptanoate (7:0), inosine, and guanosine were negatively associated with ambient temperature exposure. Heptanoate (7:0) or Heptanoic acid is a straight medium-chain saturated fatty acid that contributes to the odor of some rancid oils and used in the preparation of esters for the fragrance industry, and as an additive in cigarettes. It has a role as a plant metabolite [40]. It is capable of donating a hydron to an acceptor however little is known about health effects. On the other hand, guanosine and inosine are part of purine metabolism and have known antioxidant activity [41]. They have shown protection of DNA in-vitro from oxidative damage induced by reactive oxygen species (ROS) and served as radioprotectors in mice [41]. The negative association of ambient temperature exposure and those metabolites may indicate the potential vulnerability to oxidative stress by long-term exposure to ambient temperature.

Long-term exposures to air pollution [1–3] and temperature [4] have been linked to oxidative stress and inflammation. The identified pathways associated with long-term exposure to PM_2.5_ and temperature, similar to short-term exposure, are related to inflammation, oxidative stress, immunity, and nucleic acid damage and repair. Air pollutants may act directly as oxidants or generate free radicals that can cause oxidative stress [42]. One primary target of the reactive oxygen species derived from air pollutants is the cell membrane. Oxidative stress activates phospholipase A2 which then hydrolyzes phospholipids from the cell membrane to generate polyunsaturated free fatty acid and lyso-phospholipid [43, 44]. Due to the existence of double carbon bonds, the resulting fatty acids can be subsequently oxidized by oxygenase [44] and form products that are potential biomarkers for oxidative stress affecting lipids [45].

In our study, we observed associations with perturbed glycerophospholipid metabolism and unsaturated fatty acids biosynthesis that are main components of biological membranes as well as downstream products from oxidation of the membranes, respectively [43, 44]. This is consistent with our finding with short-term air pollution (will be reported elsewhere). Also, unsaturated fatty acids e.g., linolenic acid has been identified in association with air long-term air pollution exposure (mainly ultrafine particle (UFP)) that mediated adult onset asthma and cardio-cerebrovascular diseases [23]. We also observed perturbations in sphingolipid metabolism that is involved in inflammation and immunity [46, 47] consistent with finding with short-term air pollution [48]. Sphingolipid is also implicated in membrane biology [49] and has been linked to diabetes, hepatocellular carcinoma, and Alzheimer’s disease [50, 51], heart failure [52], and cancer [47]. We observed perturbations in glutathione metabolism, involved in xenobiotic-mediated oxidative stress [53, 54], which has been reported to be associated with traffic-related air pollution [55]. We observed perturbations in the metabolism of sulfur-containing amino acids, taurine and hypotaurine, which are readily oxidized [56]. This finding is consistent with exposure to short-term air pollution. In addition, consistent with findings with short-term air pollution, we observed perturbations in propanoate metabolism that is involved in branched-chain amino acids metabolism and the short-chain fatty acid metabolism [57]. Propanoate metabolism is involved in the colon microbiome and gluconeogenesis in the liver and acetate [58]. We observed perturbations in purine and beta-alanine metabolisms that are related to nucleic acid metabolism, damage and repair [59] consistent with reported association with air pollution, including with increased levels of 8-OHdG [19, 60]. Those metabolic signatures could be potential biomarkers for long-term exposure to air pollution and temperature. Our results need to be replicated and validated in future studies to identify the specific pathways and metabolites perturbed by air pollution and temperature exposure and thus promising ways for prevention and therapy. Our results with temperature exposure could explain our previously reported excess mortality with both high and low temperature exposure that was not explained by heat [61].

To date, only three studies focused on long-term air pollution exposure. A cross-sectional study, among a subset (*N* = 523) of the TwinsUK cohort quantified untargeted metabolomic profiling (280 metabolites) [21]. The study reported that oxidative stress and inflammation related metabolites such as α-tocopherol, glycine, and benzoate were associated with long-term PM_2.5_ and lung functions [21]. Another cross-sectional study of healthy participants (*N* = 59) measured 79 metabolites and reported associations between exposure to year-long UFP with metabolic variations related to antioxidant pathways and endothelial function [22]. An additional publication that used data from Italy and Switzerland reported that metabolic pathways e.g., Linoleate metabolism, an unsaturated fatty acid, mediated the long-term effect of air pollution on adult onset asthma and cardiovascular disease [23]. Nevertheless, our results were generally consistent with the previous literature. All other studies had smaller sample size. None of the previous studies have used ICA in the analysis, which is a new analytical approach for high dimensional and intercorrelated data and none had co-adjusted for ambient temperature. To the best of our knowledge, no studies have examined the association with long-term exposure to temperature on the metabolome.

Our results for long-term exposure to PM_2.5_ and temperature are consistent with our findings with short-term exposure in the same study population (will be reported elsewhere). Short- and long-term exposure to PM_2.5_ was associated with unique 40, and 58 metabolites, respectively and five similar perturbed metabolic pathways. On the other hand, long-term exposure to temperature was associated with six significant metabolites consistent with perturbed biosynthesis of unsaturated fatty acids, while short-term temperature exposure was associated with 40 significant metabolites and four perturbed metabolic pathways. We observed that long-term exposure to NO_2_ was associated with 15 unique significant metabolites but without significant perturbed metabolic pathways. However, short-term exposure to NO_2_ was significantly associated with 100 unique metabolites and four perturbed metabolic pathways (glycerophospholipid, glutathione, beta-alanine, and taurine and hypotaurine metabolisms). In addition, long-term exposure to O_3_ was not significantly associated with any metabolites, while short-term exposure to O_3_ was significantly associated with cysteine, which is a component of methionine, cysteine, SAM and taurine metabolism.

Our study had limitations including that a central site was used to assess the annual averages of the levels of outdoor air pollutants and temperature, which may differ from personal exposure. However, this potential misclassification may have led to underestimation of the association of interest instead of creating spurious ones [62, 63]. We acknowledge the limitation of the exposure assessment in this study, however because this is the first study to explore this association, the results will need to be confirmed or refuted in future studies. Future studies would still benefit from the insight the study may provide given the big sample size. Our study participants were older white men who lived in the greater Boston area that is a low-polluted environment that may limit the generalizability of the results. The pathway analysis was exploratory with its inherent limitation of not taking the effect direction into account. Instead, pathway analysis is based on over representation and pathway topography i.e., position of the metabolite within pathway (if it is a hub metabolite or not). However, since metabolism is complex process with feedback loops, any effects on the pathway regardless of the direction is considered perturbation and worth further investigation. The results will need to be replicated in other areas and among women and more diverse population. In this all-male study, we can only speculate on the sex differences that have been reported in the effect of temperature and less consistently on effect of air pollution. Women have more subcutaneous fat than men and this could play a role in how temperature could affect metabolome. Hormonal changes especially post-menopausal also could matter in similar ways that could affect their risks for other disease e.g. cardiovascular disease.

Finally, because the NAS is an observational study, residual confounding cannot be excluded. However, we adjusted for several confounders. In addition, the results were reassuringly consistent when using different statistical methods. Our study had also a few strengths including that the NAS study participants were geographically stable and well followed-up since they were enrolled with > 80% response rates. In addition, we quantified the largest number of untargeted metabolites to date in association with long-term air pollution that increased the statistical power. We used an untargeted global approach to quantify metabolomics which has the potential to increase metabolomic coverage and to reduce bias towards identifying well-studied metabolites [16]. To the best to our knowledge, this is the first study to report a metabolomic signature of long-term temperature exposure. Finally, we used several rigorous statistical methods including the first use of ICA for metabolomic data and we also conducted pathway analysis to facilitate the mechanistic understanding and translation of the results.

## Conclusions

Using an untargeted metabolomic approach, we identified several significant metabolites and metabolic pathways associated with long-term exposure to PM_2.5_ and temperature. We quantified the largest number of untargeted metabolites to date in association with long-term air pollution, first study to report a metabolomic signature of long-term temperature exposure, and the first to use ICA in the analysis of both. In this study, we provided insights about the metabolomic signatures of the long- and previously the short-term exposure to air pollution and temperature and the associated perturbation of the metabolic pathways. Global and untargeted metabolomic profiling is a powerful approach to increase the mechanistic understanding and the future biomarkers. Current developments of approaches for the annotation of metabolic pathways identified in the untargeted approach of the blood metabolome will allow future studies to delve deeper into the identity of specific metabolites that are affected by air pollution and temperature exposures. Our results should be replicated and validated in other studies of both short- and long-term effects of air pollution and temperature on human health. It is important in the future to identify the metabolomic signatures of the different components of PM_2.5_ to be able to identify the exposure sources.

## Supplementary Information

**Additional file 1.**

## Data Availability

The datasets used and/or analyzed during the current study are available from the corresponding author on reasonable request.
