# Supplementary material for: Metabolomic signatures of the long-term exposure to air pollution and temperature
Source: Environ Health. 2021 Jan 7;20:3. doi: 10.1186/s12940-020-00683-x (PMC7788989; doi:10.1186/s12940-020-00683-x)
Supplement: Supplementary file 1 — Additional file 1. [file 12940_2020_683_MOESM1_ESM.docx]

**Supplemental Figure 1. Volcano Plots presenting the adjusted associations between long-term (annual) exposure to air pollutants and temperature with metabolomics (single pollutant models).**

| 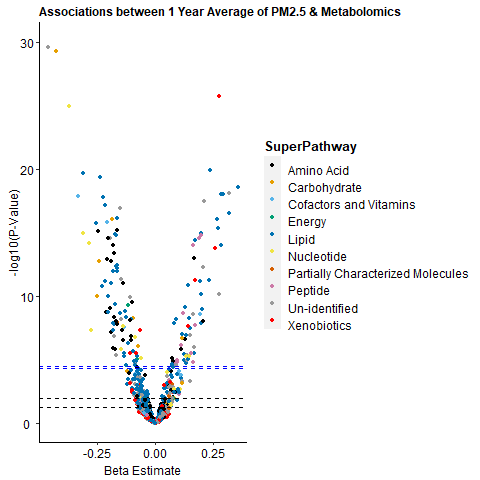 | 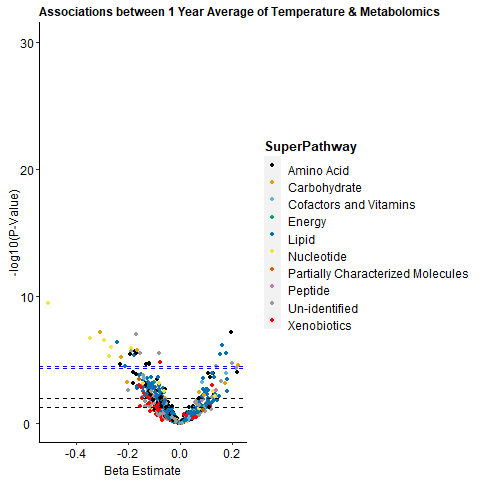 |
| --- | --- |
| 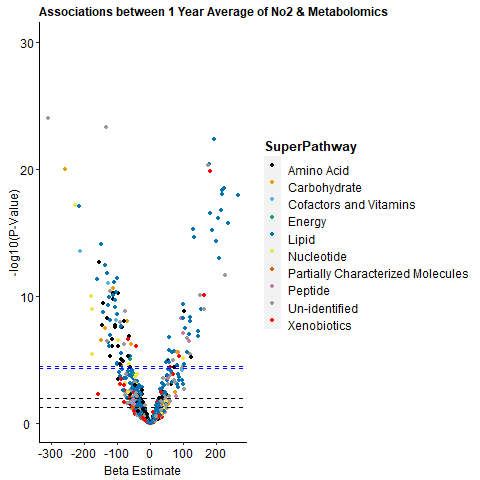 | 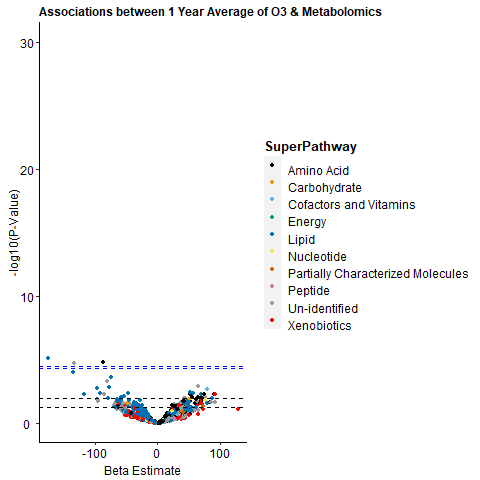 |

These models were linear mixed-effect regression models (LMEM) with random participant-specific intercepts and adjusted for PM_2.5_, NO_2_, O_3_, and temperature for each exposure window individually (single-pollutant models). The single-pollutant models for air pollutants were simultaneously adjusted for temperature.

All models were adjusted for age (years), body mass index (kg/m^2^), cigarette pack-years, alcohol intake (< or ≥2 drinks per day), socioeconomic status (income payment and years of education), season (warm/cold), and relative humidity.

The transverse dashed lines represent different statistical significance levels of p-values (from lower to upper): 0.05, 0.01, ENT95%, and ENT99%. Note the different scale of the X axes.

Abbreviations: ENT: Effective/independent number of tests; log10, logarithmic base 10; PM_2.5_, particulate matter ≤2.5 micrometers; NO_2_, nitrogen dioxide; and O_3_, ozone.

# **Supplemental Table 1. The significant associations between long-term (annual) exposure to NO2 and metabolites (multi-pollutant models) at ENT95% significance level.**

| **BIOCHEMICAL** | **super_pathway** | **SUB_PATHWAY** | **Beta (SE) for individual metabolites** | **P-value for individual metabolites** | **ICA_factor2 Weight** | **ICA_factor2 Rank** |
| --- | --- | --- | --- | --- | --- | --- |
| Cysteinylglycine | Amino Acid | Glutathione Metabolism | 226(48.0) | 5.08E-06 | 0.266 | 115 |
| Sarcosine | Amino Acid | Glycine, Serine and Threonine Metabolism | -284(41.5) | 1.09E-10 | 0.094 | 291 |
| Cysteine S-Sulfate | Amino Acid | Methionine, Cysteine, SAM and Taurine Metabolism | 358(60.5) | 1.60E-08 | 0.412 | 50 |
| Bilirubin (Z,Z) | Cofactors and Vitamins | Hemoglobin and Porphyrin Metabolism | -244(48.0) | 8.71E-07 | -0.117 | 243 |
| Azelate (C9-DC) | Lipid | Fatty Acid, Dicarboxylate | 219(38.1) | 3.70E-08 | -0.316 | 87 |
| Sebacate (C10-DC) | Lipid | Fatty Acid, Dicarboxylate | 247(44.4) | 1.00E-07 | -0.268 | 111 |
| 8-Hydroxyoctanoate | Lipid | Fatty Acid, Monohydroxy | 268(48.7) | 1.18E-07 | -0.303 | 95 |
| Caproate (6:0) | Lipid | Medium Chain Fatty Acid | 198(33.4) | 1.60E-08 | -0.155 | 197 |
| Allantoin | Nucleotide | Purine Metabolism, (Hypo)Xanthine/Inosine containing | 168(34.9) | 3.12E-06 | 0.012 | 1007 |
| HEPES | Xenobiotics | Chemical | -766(124.1) | 4.00E-09 | 0.062 | 447 |
| X - 13007 | Un-identified |  | 273(49.8) | 1.40E-07 | -0.266 | 114 |
| X - 24306 | Un-identified |  | -224(47.3) | 4.26E-06 | -0.165 | 184 |
| X - 23636 | Un-identified |  | -176(26.5) | 3.30E-10 | 0.141 | 214 |
| X - 14904 | Un-identified |  | 322(51.2) | 2.00E-09 | -0.119 | 237 |
| X - 10458 | Un-identified |  | 289(48.0) | 1.00E-08 | 0.022 | 865 |

These models were linear mixed-effect regression models (LMEM) with random participant-specific intercepts and simultaneously adjusted for PM_2.5_, NO_2_, O_3_, and temperature (multi-pollutant models) for the same exposure window.

Beta (SE) and p-values presented are from individual metabolites analysis.

All models were adjusted for age (years), body mass index (kg/m^2^), cigarette pack-years, alcohol intake (< or ≥2 drinks per day), socioeconomic status (income payment and years of education), season (warm/cold), and relative humidity.

Significant metabolites at ENT95% significance level only are presented.

ICA_factor2 rank represents the rank of the corresponding metabolite contributing to factor 2 from the independent component analysis (ICA). Higher rank (and higher weights) means higher contribution to factor 2 of the ICA. We show alongside the weights and ranks of each metabolite that contributes to factor 2 from the ICA only because it was the only significant factor.

Range of facto2- ICA rank is from 1 to 1158 i.e., number of examined metabolites. Range of facto2- ICA weight is from -1 to 1.

The metabolites with an X-XXXX format are unknown (to date Metabolon has not been able to name the metabolite) but reproducible(Metabolon is reliably able to characterize this metabolite in multiple samples and studies).

Abbreviations: ENT: Effective/independent number of tests; PM_2.5_, particulate matter ≤2.5 micrometers; NO_2_, nitrogen dioxide; and O_3_, ozone; ICA, independent component analysis; SE, standard error.

# **Supplemental Table 2. The adjusted associations between long-term exposure to air pollutants and temperature with the 5 factors of ICA (multi-pollutant models).**

|  | PM_2.5_ | | | NO_2_ | | | O_3_ | | | Temperature | | |
| --- | --- | --- | --- | --- | --- | --- | --- | --- | --- | --- | --- | --- |
| ICA Factors | **Beta (SE)** | **P-value** | **FDR P-value** | **Beta (SE)** | **P-value** | **FDR P-value** | **Beta (SE)** | **P-value** | **FDR P-value** | **Beta (SE)** | **P-value** | **FDR P-value** |
| ICA_factor1 | 0.03(0.07) | 0.68 | 0.99 | -45.18(56.71) | 0.43 | 0.99 | 19.18(40.75) | 0.64 | 0.99 | 0.03(0.06) | 0.65 | 0.99 |
| ICA_factor2 | -0.44(0.08) | 3.29E-08 | 6.40E-07 | 3.23(62.17) | 0.96 | 0.99 | -24.87(40.88) | 0.54 | 0.99 | -0.06(0.06) | 0.31 | 0.99 |
| ICA_factor3 | 0.19(0.08) | 0.02 | 0.16 | -149.24(67.21) | 0.03 | 0.28 | -60.6(46.49) | 0.19 | 0.99 | -0.09(0.07) | 0.17 | 0.99 |
| ICA_factor4 | -0.19(0.08) | 0.02 | 0.16 | 147.54(64.51) | 0.02 | 0.28 | 57.12(45.66) | 0.21 | 0.99 | -0.05(0.06) | 0.48 | 0.99 |
| ICA_factor5 | 0.02(0.07) | 0.74 | 0.99 | -69.98(52.94) | 0.19 | 0.99 | -12.32(37.69) | 0.74 | 0.99 | -0.03(0.05) | 0.63 | 0.99 |

These models were linear mixed-effect regression models (LMEM) with random participant-specific intercepts and simultaneously adjusted for PM_2.5_, NO_2_, O_3_, and temperature (multi-pollutant models) for the same exposure window.

All models were adjusted for age (years), body mass index (kg/m^2^), cigarette pack-years, alcohol intake (< or ≥2 drinks per day), socioeconomic status (income payment and years of education), season (warm/cold), and relative humidity.

ICA_factor2 rank represents the rank of the corresponding metabolite contributing to factor 2 from the independent component analysis (ICA). Higher rank (and higher weights) means higher contribution to factor 2 of the ICA. We show alongside the weights and ranks of each metabolite that contributes to factor 2 from the ICA only because it was the only significant factor.

Range of facto2- ICA rank is from 1 to 1158 i.e., number of examined metabolites. Range of facto2- ICA weight is from -1 to 1.

Abbreviations: ENT: Effective/independent number of tests; PM_2.5_, particulate matter ≤2.5 micrometers; NO_2_, nitrogen dioxide; and O_3_, ozone; ICA, independent component analysis; FDR, Benjamini-Hochberg false discovery rate; SE, standard error.

# **Supplemental Table 3. The adjusted associations between different exposure windows of air pollutants and temperature with the 5 factors of ICA (Single-pollutant models).**

|  | PM_2.5_ | | | NO_2_ | | | O_3_ | | | Temperature | | |
| --- | --- | --- | --- | --- | --- | --- | --- | --- | --- | --- | --- | --- |
| ICA factors | **Beta (SE)** | **P-value** | **FDR P-value** | **Beta (SE)** | **P-value** | **FDR P-value** | **Beta (SE)** | **P-value** | **FDR P-value** | **Beta (SE)** | **P-value** | **FDR P-value** |
| ICA_factor1 | -0.02(0.03) | 0.51 | 0.99 | -30.55(25.72) | 0.24 | 0.99 | 36.41(35.31) | 0.30 | 0.99 | -0.01(0.05) | 0.79 | 0.99 |
| ICA_factor2 | -0.44(0.04) | 0.00 | 1.22E-24 | -281.32(27.81) | 2.08E-19 | 4.16E-18 | 25.54(38.74) | 0.51 | 0.99 | -0.29(0.06) | 5.11E-06 | 0.0001 |
| ICA_factor3 | 0.03(0.04) | 0.50 | 0.99 | -10.56(29.91) | 0.72 | 0.99 | -10.49(40.04) | 0.79 | 0.99 | -0.08(0.06) | 0.18 | 0.99 |
| ICA_factor4 | -0.03(0.04) | 0.44 | 0.99 | 9.71(29.18) | 0.74 | 0.99 | 6.67(39.63) | 0.87 | 0.99 | -0.06(0.06) | 0.29 | 0.99 |
| ICA_factor5 | -0.05(0.03) | 0.08 | 0.79 | -52.83(23.87) | 0.03 | 0.28 | 15.42(32.59) | 0.64 | 0.99 | -0.07(0.04) | 0.14 | 0.99 |

These models were linear mixed-effect regression models (LMEM) with random participant-specific intercepts and adjusted for PM_2.5_, NO_2_, O_3_, and temperature for each exposure window individually (single-pollutant models). The single-pollutant models for air pollutants were simultaneously adjusted for temperature.

All models were adjusted for age (years), body mass index (kg/m^2^), cigarette pack-years, alcohol intake (< or ≥2 drinks per day), socioeconomic status (income payment and years of education), season (warm/cold), and relative humidity.

ICA_factor2 rank represents the rank of the corresponding metabolite contributing to factor 2 from the independent component analysis (ICA). Higher rank (and higher weights) means higher contribution to factor 2 of the ICA. We show alongside the weights and ranks of each metabolite that contributes to factor 2 from the ICA only because it was the only significant factor.

Range of facto2- ICA rank is from 1 to 1158 i.e., number of examined metabolites. Range of facto2- ICA weight is from -1 to 1.

Abbreviations: ENT: Effective/independent number of tests; PM_2.5_, particulate matter ≤2.5 micrometers; NO_2_, nitrogen dioxide; and O_3_, ozone; ICA, independent component analysis; FDR, Benjamini-Hochberg false discovery rate; SE, standard error.

# **Supplemental Table 4. Metabolisms resulted of the pathway analysis of the significant metabolites associated with long-term exposure to with air pollutants and temperature.**

| Long-term PM_2.5_ exposure pathway analysis | Total | Expected | Hits | Raw P-value | -log (P) | FDR P-value | Impact |
| --- | --- | --- | --- | --- | --- | --- | --- |
| Glycerophospholipid metabolism | 10 | 3.02 | 7 | 0.009 | 4.68 | 0.78 | 0.46 |
| Propanoate metabolism | 3 | 0.91 | 3 | 0.03 | 3.63 | 0.99 | 0.04 |
| Sphingolipid metabolism | 10 | 3.02 | 6 | 0.04 | 3.11 | 0.99 | 0.26 |
| Glutathione metabolism | 7 | 2.11 | 4 | 0.12 | 2.08 | 0.99 | 0.07 |
| beta-Alanine metabolism | 8 | 2.41 | 4 | 0.19 | 1.64 | 0.99 | 0.40 |
| Purine metabolism | 12 | 3.62 | 5 | 0.28 | 1.28 | 0.99 | 0.23 |
| Glycosylphosphatidylinositol (GPI)-anchor biosynthesis | 1 | 0.30 | 1 | 0.30 | 1.20 | 0.99 | 0.004 |
| Cysteine and methionine metabolism | 11 | 3.32 | 4 | 0.44 | 0.83 | 0.99 | 0.10 |
| Pyrimidine metabolism | 11 | 3.32 | 4 | 0.44 | 0.83 | 0.99 | 0.17 |
| Butanoate metabolism | 5 | 1.51 | 2 | 0.48 | 0.74 | 0.99 | 0 |
| Nicotinate and nicotinamide metabolism | 5 | 1.51 | 2 | 0.48 | 0.74 | 0.99 | 0.19 |
| Porphyrin and chlorophyll metabolism | 5 | 1.51 | 2 | 0.48 | 0.74 | 0.99 | 0.21 |
| Taurine and hypotaurine metabolism | 5 | 1.51 | 2 | 0.48 | 0.74 | 0.99 | 0.71 |
| Ether lipid metabolism | 2 | 0.60 | 1 | 0.51 | 0.67 | 0.99 | 0 |
| Linoleic acid metabolism | 2 | 0.60 | 1 | 0.51 | 0.67 | 0.99 | 0 |
| alpha-Linolenic acid metabolism | 2 | 0.60 | 1 | 0.51 | 0.67 | 0.99 | 0 |
| Phosphatidylinositol signaling system | 2 | 0.60 | 1 | 0.51 | 0.67 | 0.99 | 0.002 |
| Retinol metabolism | 2 | 0.60 | 1 | 0.51 | 0.67 | 0.99 | 0.24 |
| Pantothenate and CoA biosynthesis | 9 | 2.72 | 3 | 0.55 | 0.61 | 0.99 | 0.02 |
| Arginine biosynthesis | 11 | 3.32 | 3 | 0.70 | 0.36 | 0.99 | 0.14 |
| Arginine and proline metabolism | 12 | 3.62 | 3 | 0.76 | 0.27 | 0.99 | 0.20 |
| Arachidonic acid metabolism | 4 | 1.21 | 1 | 0.77 | 0.27 | 0.99 | 0 |
| Glycerolipid metabolism | 4 | 1.21 | 1 | 0.77 | 0.27 | 0.99 | 0.01 |
| Starch and sucrose metabolism | 4 | 1.21 | 1 | 0.77 | 0.27 | 0.99 | 0.07 |
| Pentose and glucuronate interconversions | 4 | 1.21 | 1 | 0.77 | 0.27 | 0.99 | 0.08 |
| Citrate cycle (TCA cycle) | 5 | 1.51 | 1 | 0.84 | 0.18 | 0.99 | 0.03 |
| Valine, leucine and isoleucine biosynthesis | 7 | 2.11 | 1 | 0.92 | 0.08 | 0.99 | 0 |
| Glycine, serine and threonine metabolism | 13 | 3.93 | 2 | 0.94 | 0.06 | 0.99 | 0.09 |
| Alanine, aspartate and glutamate metabolism | 13 | 3.93 | 2 | 0.94 | 0.06 | 0.99 | 0.22 |
| Lysine degradation | 8 | 2.42 | 1 | 0.94 | 0.05 | 0.99 | 0 |
| Histidine metabolism | 8 | 2.42 | 1 | 0.94 | 0.05 | 0.99 | 0 |
| Primary bile acid biosynthesis | 12 | 3.62 | 1 | 0.99 | 0.01 | 0.99 | 0.008 |
| Aminoacyl-tRNA biosynthesis | 19 | 5.74 | 2 | 0.99 | 0.008 | 0.99 | 0 |

| Long-term No2 exposure pathway analysis | Total | Expected | Hits | Raw P-value | -log(P) | FDR P-value | Impact |
| --- | --- | --- | --- | --- | --- | --- | --- |
| Ether lipid metabolism | 2 | 0.24 | 1 | 0.22 | 1.50 | 0.99 | 0 |
| D-Glutamine and D-glutamate metabolism | 3 | 0.36 | 1 | 0.32 | 1.15 | 0.99 | 0 |
| Pyrimidine metabolism | 11 | 1.31 | 2 | 0.38 | 0.96 | 0.99 | 0.09 |
| Arginine biosynthesis | 11 | 1.31 | 2 | 0.38 | 0.96 | 0.99 | 0.23 |
| Butanoate metabolism | 5 | 0.59 | 1 | 0.47 | 0.75 | 0.99 | 0 |
| Porphyrin and chlorophyll metabolism | 5 | 0.59 | 1 | 0.47 | 0.75 | 0.99 | 0.05 |
| Citrate cycle (TCA cycle) | 5 | 0.59 | 1 | 0.47 | 0.75 | 0.99 | 0.06 |
| Taurine and hypotaurine metabolism | 5 | 0.59 | 1 | 0.47 | 0.75 | 0.99 | 0.29 |
| Glutathione metabolism | 7 | 0.83 | 1 | 0.59 | 0.52 | 0.99 | 0.06 |
| Glycerophospholipid metabolism | 10 | 1.19 | 1 | 0.73 | 0.32 | 0.99 | 0.05 |
| Cysteine and methionine metabolism | 11 | 1.31 | 1 | 0.76 | 0.27 | 0.99 | 0.02 |
| Alanine, aspartate and glutamate metabolism | 13 | 1.54 | 1 | 0.82 | 0.20 | 0.99 | 0.05 |
| Glycine, serine and threonine metabolism | 13 | 1.54 | 1 | 0.82 | 0.20 | 0.99 | 0.09 |

| Long-term temperature exposure pathway analysis | Total | Expected | Hits | Raw P-value | -log(P) | FDR P-value | Impact |
| --- | --- | --- | --- | --- | --- | --- | --- |
| Biosynthesis of unsaturated fatty acids | 9 | 1.07 | 4 | 0.01 | 4.34 | 0.99 | 0 |
| Fructose and mannose metabolism | 2 | 0.24 | 1 | 0.22 | 1.50 | 0.99 | 0 |
| Amino sugar and nucleotide sugar metabolism | 2 | 0.24 | 1 | 0.22 | 1.50 | 0.99 | 0 |
| alpha-Linolenic acid metabolism | 2 | 0.24 | 1 | 0.22 | 1.50 | 0.99 | 0 |
| Linoleic acid metabolism | 2 | 0.24 | 1 | 0.22 | 1.50 | 0.99 | 1 |
| Pyruvate metabolism | 3 | 0.36 | 1 | 0.32 | 1.15 | 0.99 | 0 |
| Arachidonic acid metabolism | 4 | 0.48 | 1 | 0.40 | 0.92 | 0.99 | 0.31 |
| Purine metabolism | 12 | 1.43 | 2 | 0.43 | 0.84 | 0.99 | 0.002 |
| Citrate cycle (TCA cycle) | 5 | 0.59 | 1 | 0.47 | 0.75 | 0.99 | 0.03 |
| Porphyrin and chlorophyll metabolism | 5 | 0.59 | 1 | 0.47 | 0.75 | 0.99 | 0.07 |
| Tryptophan metabolism | 5 | 0.59 | 1 | 0.47 | 0.75 | 0.99 | 0.10 |
| Alanine, aspartate and glutamate metabolism | 13 | 1.54 | 2 | 0.47 | 0.75 | 0.99 | 0.09 |
| Galactose metabolism | 6 | 0.71 | 1 | 0.54 | 0.62 | 0.99 | 0 |
| Tyrosine metabolism | 9 | 1.07 | 1 | 0.69 | 0.37 | 0.99 | 0.02 |
| Arginine biosynthesis | 11 | 1.31 | 1 | 0.76 | 0.27 | 0.99 | 0 |
| Pyrimidine metabolism | 11 | 1.31 | 1 | 0.76 | 0.27 | 0.99 | 0.06 |
| Arginine and proline metabolism | 12 | 1.43 | 1 | 0.79 | 0.23 | 0.99 | 0.01 |
| Glycine, serine and threonine metabolism | 13 | 1.54 | 1 | 0.82 | 0.20 | 0.99 | 0 |

| ICA_factor2_highest weights100 pathway analysis | Total | Expected | Hits | Raw P-value | -log(P) | FDR P-value | Impact |
| --- | --- | --- | --- | --- | --- | --- | --- |
| Purine metabolism | 12 | 2.44 | 6 | 0.02 | 4.05 | 0.99 | 0.23 |
| Sphingolipid metabolism | 10 | 2.03 | 5 | 0.03 | 3.47 | 0.99 | 0.26 |
| beta-Alanine metabolism | 8 | 1.62 | 4 | 0.06 | 2.90 | 0.99 | 0.06 |
| Glycerophospholipid metabolism | 10 | 2.03 | 4 | 0.12 | 2.1 | 0.99 | 0.19 |
| Starch and sucrose metabolism | 4 | 0.81 | 2 | 0.18 | 1.69 | 0.99 | 0.49 |
| Neomycin, kanamycin and gentamicin biosynthesis | 1 | 0.20 | 1 | 0.20 | 1.59 | 0.99 | 0 |
| Glycosylphosphatidylinositol (GPI)-anchor biosynthesis | 1 | 0.20 | 1 | 0.20 | 1.59 | 0.99 | 0.004 |
| Nicotinate and nicotinamide metabolism | 5 | 1.01 | 2 | 0.27 | 1.32 | 0.99 | 0.19 |
| Porphyrin and chlorophyll metabolism | 5 | 1.01 | 2 | 0.27 | 1.32 | 0.99 | 0.23 |
| Taurine and hypotaurine metabolism | 5 | 1.01 | 2 | 0.27 | 1.32 | 0.99 | 0.71 |
| Galactose metabolism | 6 | 1.22 | 2 | 0.35 | 1.04 | 0.99 | 0.03 |
| Fructose and mannose metabolism | 2 | 0.41 | 1 | 0.37 | 1.01 | 0.99 | 0 |
| Amino sugar and nucleotide sugar metabolism | 2 | 0.41 | 1 | 0.37 | 1.01 | 0.99 | 0 |
| Glutathione metabolism | 7 | 1.42 | 2 | 0.43 | 0.84 | 0.99 | 0.01 |
| Propanoate metabolism | 3 | 0.61 | 1 | 0.50 | 0.70 | 0.99 | 0.04 |
| Histidine metabolism | 8 | 1.62 | 2 | 0.51 | 0.68 | 0.99 | 0.09 |
| Pantothenate and CoA biosynthesis | 9 | 1.83 | 2 | 0.58 | 0.55 | 0.99 | 0 |
| Tryptophan metabolism | 5 | 1.01 | 1 | 0.68 | 0.38 | 0.99 | 0.10 |
| Arginine biosynthesis | 11 | 2.23 | 2 | 0.69 | 0.37 | 0.99 | 0.08 |
| Arginine and proline metabolism | 12 | 2.44 | 2 | 0.74 | 0.30 | 0.99 | 0.09 |
| Valine, leucine and isoleucine biosynthesis | 7 | 1.42 | 1 | 0.80 | 0.22 | 0.99 | 0 |
| Cysteine and methionine metabolism | 11 | 2.23 | 1 | 0.92 | 0.08 | 0.99 | 0.06 |
| Pyrimidine metabolism | 11 | 2.23 | 1 | 0.92 | 0.08 | 0.99 | 0.07 |
| Aminoacyl-tRNA biosynthesis | 19 | 3.86 | 2 | 0.93 | 0.075 | 0.99 | 0 |
| Primary bile acid biosynthesis | 12 | 2.44 | 1 | 0.94 | 0.065 | 0.99 | 0.008 |
| Glycine, serine and threonine metabolism | 13 | 2.64 | 1 | 0.95 | 0.05 | 0.99 | 0 |
| Alanine, aspartate and glutamate metabolism | 13 | 2.64 | 1 | 0.95 | 0.05 | 0.99 | 0.22 |

Abbreviations: PM_2.5_, particulate matter ≤2.5 micrometers; NO_2_, nitrogen dioxide; and O_3_, ozone; ICA, independent component analysis; FDR, Benjamini-Hochberg false discovery rate.

Total is the total number of compounds in the pathway; the Hits is the actually matched number from the data; the Raw p is the original p-value calculated from the enrichment analysis; the FDR p is the p-value adjusted using False Discovery Rate; the Impact is the pathway impact value calculated from pathway topology analysis.

-Log (P) here denotes the minus of the natural log of the raw p-value.
